# Online Information Related to Symptoms of Carpal Tunnel Syndrome: A Google Search Analysis

**DOI:** 10.7759/cureus.35586

**Published:** 2023-02-28

**Authors:** Brian K Foster, Nicholas R Brule, Clarice Callahan, Jessica Baylor, Joel C Klena, Louis C Grandizio

**Affiliations:** 1 Orthopaedics, Geisinger Medical Center, Danville, USA; 2 Orthopaedics, Geisinger Commonwealth School of Medicine, Danville, USA

**Keywords:** hand surgery, patient education, google, median nerve, carpal tunnel syndrome

## Abstract

Introduction

While Google is frequently used to access internet-based health resources, the quality of online health information remains variable. Our purpose was to assess suggested resources identified through Google search features for common symptoms related to carpal tunnel syndrome (CTS).

Methods

Two searches were performed. The first, labeled "symptom-related," included the terms "hand numbness," "hand tingling," and "hand falling asleep." The second, labeled "CTS-specific," included "carpal tunnel syndrome," "carpal tunnel surgery," and "carpal tunnel release." A novel feature of Google’s search engine is to display similar searches made by other users ("People Also Ask" snippet). For each search, the first 100 results snippets and the associated website links were recorded. A list of unique questions was compiled and classified into 1 of 3 categories using the Rothwell classification: fact, policy, or value. Questions were also classified based on the diagnoses suggested by the query. Website authorship was determined, and the corresponding links were categorized by two independent reviewers.

Results

The "symptom-related" searches yielded 175 unique questions and 130 unique website links, and the "CTS-specific" searches yielded a total of 243 questions and 179 unique links. For "symptom-related" searches, 65% of questions suggested a diagnosis, with CTS being suggested as a diagnosis for only 3% of questions. In contrast, CTS was suggested by 92% of questions in "CTS-specific" searches. In both searches, nearly 75% of questions were classified as "facts." Commercial websites were the most common in both searches.

Conclusion

Google searches for common symptoms of median nerve compression rarely yield information related to CTS.

## Introduction

The landscape of how patients obtain information regarding medical conditions and treatments has changed rapidly in the 21st century [1,2]. Patient use of the internet to learn about medical conditions has become almost ubiquitous in recent years [2,3]. Approximately 75% of adults in the United States use the internet as their first resource when seeking information for health-related conditions [3]. However, the reliability of online health information continues to be questioned. Within upper-extremity surgery, prior investigations have suggested that anywhere from 20% to 50% of sites with health information may be misleading and often reinforce common misconceptions [4-8]. Furthermore, patients often have difficulty finding helpful information, with reports that patients easily find the information they seek less than 50% of the time [3].

Carpal tunnel syndrome (CTS) is the most common compressive neuropathy of the upper extremity, affecting one to three patients per 1,000 per year [9]. Costs associated with the care of CTS exceed two billion dollars annually [9]. The quality of online patient information regarding CTS is particularly concerning [4-6]. A novel feature of Google’s search engine is the "People Also Ask" snippet, where similar searches by other users are displayed. These serve to guide patients to related topics and provide a single website link with relevant information. Recent studies involving both shoulder and lower extremity arthroplasty examined these "frequently asked questions" and found that information came from a variety of sources and provided insights into what patients were viewing online [10,11]. The results of this Google search feature, as it pertains to CTS or symptoms of median nerve compression, remain unknown.

The purpose of this investigation was to quantify and assess suggested resources identified through Google search features for common symptoms related to CTS as well as for direct searches regarding CTS and related surgery. In addition, we aimed to characterize the suggested resources provided by Google’s "People Also Ask" snippet. We hypothesized that suggested information found using symptom-related search terms would be related to CTS and would be predominantly produced by academic centers.

## Materials and methods

Two groups of searches were performed. The first, labeled "symptom-related," included the terms "hand numbness," "hand tingling," and "hand falling asleep." The second, labeled "CTS-specific," included "carpal tunnel syndrome," "carpal tunnel surgery," and "carpal tunnel release." Each term was individually queried into a Google Web Search (www.google.com) that was clean-installed with the browser history removed to eliminate bias from Google’s personalized search algorithm. The first search was performed in September 2021, and the second search was performed in December 2022. For each search, the first 100 results of the "People Also Ask" snippet and the associated website links were recorded. A list of unique questions was compiled. Each question was categorized, according to Rothwell’s classification, into fact (is the given question true?) or policy (will a course of action solve a specific problem?) or value (is an idea, object, or event good or bad?) [12].

Questions populated by the "People Also Ask" snippet were classified based on any diagnosis suggested by the question, post hoc. These categories included cardiovascular, psychological, diabetes, nutrition, hydration, multiple sclerosis, nerve injury/damage, carpal tunnel syndrome, COVID-19, and none for the searches related to symptoms of carpal tunnel. Similar to previous studies [10,11], each website was also categorized by site authorship/ownership. These categories included commercial, academic, medical practice, single surgeon practice, government, and social media.

The questions and the corresponding links were categorized by two independent reviewers. Interobserver reliability was calculated using Cohen’s kappa coefficient. Discrepancies were resolved via consensus discussion with the senior author, where the category was discussed amongst all parties and the final decision was made by consensus regarding the definitive category. When discussing agreement levels for Cohen’s kappa coefficient, the following guidelines were utilized: slight (0.01-0.20), fair (0.21-0.40), moderate (0.41-0.60), substantial (0.61-0.81), and near perfect (>0.81) [13,14].

## Results

The "symptom-related" searches yielded a total of 230 unique questions and 130 unique website links for review. Fifty-five (24%) questions were deemed irrelevant and excluded, leaving 175 questions for evaluation. Irrelevant questions often related to common suggested diagnoses but did not allude to any of the queried symptoms. Cohen’s kappa coefficient was 0.954 for question classification and 1.000 for website classification.

The "CTS-specific" searches yielded a total of 243 unique questions and 179 unique links for review. No questions were deemed irrelevant. Cohen’s kappa coefficient is 0.815 for question classification and 0.844 for website classification.

**Figure 1:**
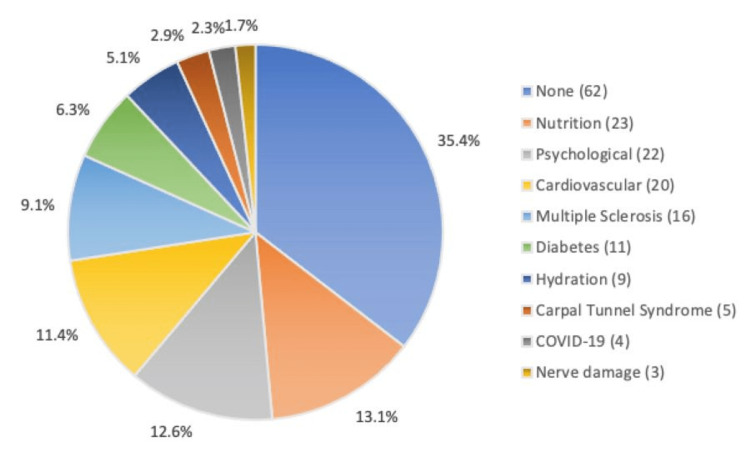
Common diagnoses as suggested through Google’s “People Also Ask” snippet for queries of “symptom-related” searches.

For "symptom-related" searches, a diagnosis was suggested in 65% (120/175) of questions (Figure 1). The most common suggested diagnosis was nutrition-based (13%, n=23), followed by psychological (12%, n=22) and cardiovascular (11%, n=20). CTS and nerve damage/injury were suggested in 3% (n=5) and 2% (n=3) of questions, respectively.

**Figure 2:**
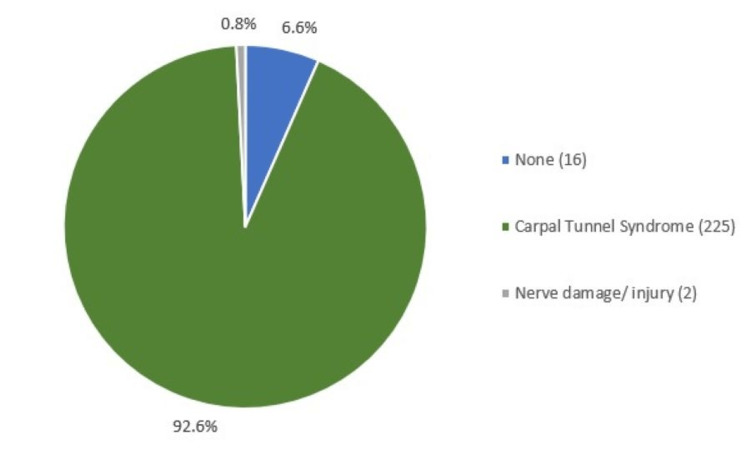
Common diagnoses as suggested through Google’s “People Also Ask” snippet for queries of “CTS-specific” searches.

For "CTS-specific" searches, a diagnosis was suggested in 93% (227/243) of questions (Figure 2). CTS was the most commonly suggested diagnosis, occurring in 92% (n=225) of the questions. Two questions (0.82%) were related to nerve damage/injury.

For "symptom-related" searches, 75% (n=131) of questions were classified as fact questions (Table 1). Value questions represented 20% (n=35) of search questions, and policy questions represented 5.1% (n=9). The most common website categories were commercial (53.1%, n=69), followed by academic (22.3%, n=29), and medical practice (17.7%, n=23) (Table 2).

**Table 1:** Rothwell’s classification of questions, question classification by suggested diagnosis, and website categorization.

Rothwell’s classification	Description
Fact	Asks whether something is true and to what extent Example: Can dehydration cause tingling?
Policy	Asks whether a specific course of action should be taken to solve a problem. Example: When should you see a neurologist for numbness?
Value	Asks for evaluation of an idea, object, or event. Example: Is it bad if your left hand goes numb?
Suggested diagnosis	
Cardiovascular	Includes questions about circulation, blood pressure, and stroke
Psychological	Includes questions about stress and anxiety, Example: Can stress cause numbness in hands?
Diabetes	Includes any reference to blood sugar - high or low
Nutrition	Includes vitamin, mineral, or any other dietary deficiency and/or excess. Example: Can vitamin D cause tingling?
Hydration	Includes any reference towards hydration status. Example: Can dehydration cause numbness?
Multiple sclerosis	Includes questions related to diagnosis and symptoms of multiple sclerosis
Nerve damage	Includes any question explicitly mentioning nerve damage. Example: What are the signs of nerve damage in your hands?
Carpal tunnel syndrome	Includes any question explicitly mentioning carpal tunnel. Example: How can I check myself for carpal tunnel?
COVID	Includes any question explicitly mentioning COVID. Example: Is tingling a symptom of COVID?
Website categorization	
Commercial	Organizations that provide public health information, including medical device/manufacturing/pharmaceutical companies and news outlets. Example: WebMD, Healthline.
Academic	Universities, academic medical centers, or academic societies. Example: AAOS, hopkinsmedicine.org, public and private university hospitals.
Medical practice	Local hospitals or medical groups without clear academic affiliation. Example: Boston Shoulder Institute.
Government	Websites maintained by a national government. Example: MedlinePlus, PubMed.
Social media	Blogs, internet forums, support groups, and nonmedical organizations primarily designed for information and video sharing. Example: Facebook.

**Table 2:** Rothwell’s classification of questions and website categorization.

Rothwell's classification	Count	Percent
Fact	131	75%
Value	35	20%
Policy	9	5%
Website categorization		
Commercial	69	53%
Academic	29	22%
Medical Practice	23	18%
Social Media	7	5%
Government	2	2%

For "CTS-specific" searches, 72% (n=176) of questions were classified as fact questions. Value questions represented 14.4% (n=35) of search questions, and policy questions represented 13.17% (n=32). The most common website categories were commercial (30.7%, n=55), followed by medical practice (27.9%, n=50) and academic (18.4%, n=33).

## Discussion

Contrary to our hypothesis, search terms representing the classic symptoms of CTS do not commonly yield resources pertaining to CTS. While nearly two-thirds of FAQs alluded to a diagnosis, less than 5% were related to CTS. Other common causes of these symptoms within orthopedics, such as radiculopathy or other compressive peripheral neuropathies, were not represented at all. The more commonly recommended diagnoses from this study, such as nutritional deficiencies, dehydration, and diabetes, rarely present with hand numbness [15-17]. Most suggested questions were fact-based, which is in alignment with previous studies [10,11]. Unlike previous studies, policy questions were less common in the present study, likely representing a fundamental difference in searches seeking information on symptoms compared to those examining surgical procedures.

For "symptom-related" searches, over half of all recommended questions were associated with a commercial link. Similarly, commercial websites were the most common category for "CTS-specific" searches. These findings are in contrast to previous studies on Google’s "People Also Ask" snippet [10,11]. Sudah et al. found that academic centers were the most common source of information, with commercial sources providing only 18% of links [11]. Shen et al. similarly noted academic centers as the most common source of information, but commercial sites were the second most common source, accounting for 30% of links [10]. However, previous studies investigating online resources for CTS found that commercial websites were the most common source of information over a 15-year time period [4,5]. While the authors reported a high rate of misleading information, they did not compare this to other website categories. Other studies have noted a decreased quality of commercial websites [18,19]. Although prioritized by search engines, patients should be aware of the limitations of health information from commercially sponsored websites. Given our search findings, it is reasonable to advise patients to use caution when utilizing Google’s "People Also Ask" tool, as it may not be helpful or reliable for patients seeking health information online related to common CTS symptoms or CTS itself.

These data highlight the limitations of patient-driven searches for online health information. The issue remains that resources from academic centers and professional societies are not prioritized by Google’s search engine, as evidenced by two different search queries. Patients searching online highly value convenience when selecting websites to review [20]. Therefore, organizations such as the American Society for Surgery of the Hand and the American Academy of Orthopaedic Surgeons, with a vested interest in producing and maintaining high-quality educational resources, should work to optimize their online presence to better reach their patients.

Several limitations must be considered when interpreting our results. First, while we selected our "symptom-related" search terms based on common symptoms of CTS, our terms may not be representative of all common search terms used by patients. For example, we used the term "hand numbness" but not "why are my hands numb." Also, while Google is the most common search engine [21], our results may not be generalizable to other search engines. Because Google’s search algorithm is continually updated and changing, our results may not be generalizable over time. Google’s search algorithm also relies on a user’s previous search history, which may lead to greater variation in search results. Lastly, the impact of the suggested FAQs and associated websites on patients’ understanding and decision-making remains unknown.

## Conclusions

Google searches for common symptoms of median nerve compression rarely yield information related to CTS. Information produced by academic institutions and professional societies is infrequently accessed via these searches, and the suggested information has a strong commercial influence. Suggested FAQs by Google may not be a reliable source for health information.
